# Structured Sequence Learning: Animal Abilities, Cognitive Operations, and Language Evolution

**DOI:** 10.1111/tops.12444

**Published:** 2019-07-29

**Authors:** Christopher I. Petkov, Carel ten Cate

**Affiliations:** ^1^ Newcastle University Medical School; ^2^ Institute of Biology Leiden University

**Keywords:** Humans, Birds, Rodents, Primates, Artificial grammar learning, Cognition, Language, Combinatorial

## Abstract

Human language is a salient example of a neurocognitive system that is specialized to process complex dependencies between sensory events distributed in time, yet how this system evolved and specialized remains unclear. Artificial Grammar Learning (AGL) studies have generated a wealth of insights into how human adults and infants process different types of sequencing dependencies of varying complexity. The AGL paradigm has also been adopted to examine the sequence processing abilities of nonhuman animals. We critically evaluate this growing literature in species ranging from mammals (primates and rats) to birds (pigeons, songbirds, and parrots) considering also cross‐species comparisons. The findings are contrasted with seminal studies in human infants that motivated the work in nonhuman animals. This synopsis identifies advances in knowledge and where uncertainty remains regarding the various strategies that nonhuman animals can adopt for processing sequencing dependencies. The paucity of evidence in the few species studied to date and the need for follow‐up experiments indicate that we do not yet understand the limits of animal sequence processing capacities and thereby the evolutionary pattern. This vibrant, yet still budding, field of research carries substantial promise for advancing knowledge on animal abilities, cognitive substrates, and language evolution.

## 1 Introduction

How language evolved may always be mired in uncertainty. Much of the empirical evidence we would want to assess is missing or unavailable for study: Fossil and genetic analysis of ancestors provides important but incomplete information needed to infer cognitive abilities. Extant animals available for study differ in derived abilities, requiring data from more than the usual range of species to infer which abilities are shared via a common ancestor and which arose by way of convergent evolution or as unique specializations in certain species. Such a broad cross‐species approach is important to strive for but difficult to achieve because complex behavior is inherently variable and challenging to assess in the same way across the species. Thus, it is not unexpected that controversy abounds on how language evolved. Nonetheless, the view that the evolutionary roots of language syntax can be inferred by better assessing animal combinatorial capacities is broadly shared (Fitch et al., 2012; Petkov & Wilson, 2012; Jackendoff & Wittenberg, 2017; Schlenker et al., 2016). These capacities need not have evolved primarily for vocal communication, so we can look beyond an animal’s vocal production ability and ask about the extent of its combinatorial sequence learning capacity.

In this paper, we focus on the learning of sequences containing dependencies between items next to each other or separated by other items in time, as well as more complex, hierarchically organized dependencies. Studying sequential or serial learning has always been a prime research area in comparative cognitive science. We will tread lightly on this literature, focusing instead on the complexity of sequencing learning to ask: What is the extent of animal‐structured sequence learning ability? Do species differ in these abilities? What cognitive substrates are likely to be involved?

## 2 Artificial Grammar Learning (AGL) paradigms

**Figure 1:**
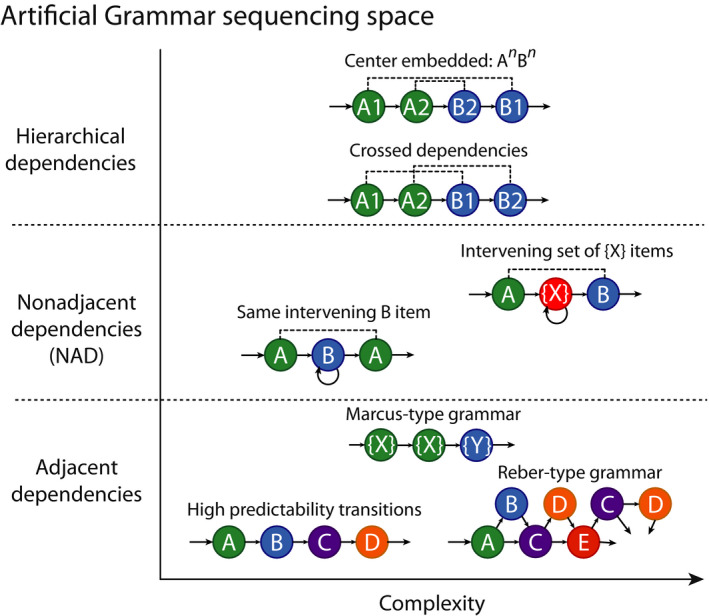
Multi‐dimensional AG sequencing complexity space. Relationships between events in a sequence can vary in complexity along multiple dimensions. *Y*‐axis defines categorical distinctions of adjacent, nonadjacent, and hierarchical. Different AGs referred to in the text are presented as state transition diagrams. A, B, C, etc. stand for specific items and X and Y for sets of items. Following the arrows generates legal sequences consistent with the AG rule(s). Deviations create “ungrammatical” or violation sequences. Within each category there are more variants than could be indicated here. Cognitive demands increase with the level of generalization (e.g., from acoustical to relational similarities among items), category set size, item numbers, etc.

The use of artificial grammars is an important approach for studying the comparative biology of language learning. In AGL experiments, participants hear or see meaningless auditory or visual items arranged in sequences (strings) generated by particular rules. The Artificial Grammar (AG) rules are typically displayed with state‐transition diagrams (Fig. 1), which define the ordering relationships between the items. AGs can emulate the ordering dependencies in syntactic or phonotactic relationships, such as adjacent or nonadjacent dependencies between syllables, words, or phrases (Santolin & Saffran, 2017). After exposure to a subset of legal AG sequences, the participants are tested with new “grammatical” and “ungrammatical” (i.e., AG “consistent” or “inconsistent”/“violation”) sequences. Responses to the different sequences are measured to assess which aspects of the ordering relationships the participants can detect.

In adult human AGL studies, the participants are typically tested by asking whether the sequence they heard or saw is similar to or differs from sequences experienced during the previous learning phase. Infants and nonhuman animals cannot be instructed in this way, so studies with such participants often use habituation/dishabituation methods in which the infant or nonhuman animal is exposed to a string of sounds organized according to the AG. A difference in the responses toward novel AG‐consistent versus violation sequences provides information into what the individual learned about the sequences. Operant training is another frequently used method in studies of nonhuman animals (ten Cate & Okanoya, 2012). With this approach, the subjects are first trained to discriminate different sets of sequences. They are next tested with new sets during probe trials that are either unrewarded or equally rewarded, to test what the animals learned about the strings and whether they generalize the learned sequencing dependencies to new sequences or items.

In this paper, we subcategorize different types of sequencing operations by variation on different dimensions and in approximate levels of sequencing complexity (Fig. 1; see also Dehaene, Meyniel, Wacongne, Wang, & Pallier, 2015; Gervain & Mehler, 2010; Gervain, delaCruz‐Pavia, & Gerken, 2018; Jaeger & Rogers, 2012; Wilson et al., 2017). With complexity, we mean variation from highly predictable adjacent sequencing dependencies to those that are less predictable, further separated in time, contain multiple nonadjacent dependencies, or are based on relational and not physical similarities. However, our understanding of the cognitive processes or mechanisms involved remains tentative. Thus, our characterization of sequencing complexity is primarily meant to inspire exploration of this multidimensional space.

Next, each section briefly overviews seminal infant studies that motivated studies of nonhuman animals. This is followed by critical assessment of animal sequence‐processing abilities from representative studies.

## 3 Adjacent dependencies: Transitional probabilities

Children segment speech streams often without obvious acoustical cues to identify word boundaries. Saffran et al. (1996) in seminal statistical learning work proposed that young children can extract statistical cues whenever these are available. These assist the child in segmenting continuous streams of, for example, nonsense syllables, for example, “bi‐da‐ku‐pa‐do‐ti‐go‐la‐bi‐da‐ku…”. Statistical regularities were created by randomly combining three specific syllables in a sequence, for example, “bi‐da‐ku," to create several “words." The transitional probabilities (TPs) between any two syllables within the words were highly predictable. For example, “bi” was always followed by “da” and then “ku." However, the transitions *between* words were less predictable. After exposure to the syllable stream, 8‐month‐old infants looked longer to both nonwords and part‐words than to the statistically predictable words. The initial study could not address whether the infants were attending to the more frequent co‐occurrence of items forming words than items forming part‐words or whether the infants relied on the reduced transitional probabilities. A later experiment (Aslin et al., 1998) showed that infants can rely on the lower transitional probabilities between words, when the co‐occurrence of syllables is controlled for.

### 3.1 Animals

The infant work (Saffran et al., 1996) motivated an experiment in which cotton‐top tamarins were exposed to the same strings (Hauser et al., 2001). After exposure, the tamarins oriented more to part‐words and nonwords than to words. However, whether the animals detected the drop in transitional probabilities between words and/or the predictable co‐occurrence of syllables is unknown.

Rats were also tested with the stimuli from the infant study (Toro & Trobalón, 2005), using a habituation paradigm. During testing, the rats discriminated between words and part‐words or nonwords. However, unlike the infants, the rats primarily relied on syllable co‐occurrence.

Songbirds, like zebra finches and starlings, can discriminate linear strings in which the song units are arranged in different orders by using both ordinal position and item sequence (Chen & ten Cate, 2015; Comins & Gentner, 2010). Whether birds can also detect “word‐like” equivalents in strings was examined by habituating zebra finches to streams of synthesized tones, of which a series of six formed a “word." Neurophysiological recordings of auditory forebrain areas CLM and NCM (Lu & Vicario, 2014) showed a differential response to the equivalents of words compared to part‐words and nonwords. In another study, zebra finches were trained to distinguish two strings of song elements which consisted of triplet analogues to words (Spierings et al., 2015). One group of birds was trained with slightly longer silent intervals between the triplets than between the elements within a triplet. Only the birds from this group showed a better recognition of element triplets equivalent to words, than those equivalent to part‐words and nonwords, suggesting that they recognized the co‐occurring items if triplets are defined by an acoustical cue such as silence. The experiments allow no conclusions on whether the birds can rely on both high and low transitional probabilities.

### 3.2 Summary

The ability to detect regularities within strings of items is broadly present. Infants can identify combinations of items (“words”) based on high or low transitional probabilities among items (e.g., Gervain et al., 2018). Nonhuman animals can detect dependencies based on co‐occurrences between elements, but whether they can also rely on low probability transitions needs further exploration. Moreover, for both human and nonhuman animals positional information or prosodic cues such as silent gaps between syllables can greatly assist speech stream segmentation (Mueller et al., 2018).

## 4 Adjacent dependencies: Variable sequences

Reber (1967) tested adult humans using rule‐based sequences where the statistical cues become more variable and several items transition to one or more others. “Reber‐type” paradigms have been relied on to assess how well infants manage with sequences of varying length and transitional probabilities (see Fig. 1), often using nonsense word strings, for example, “pel," “vot," “tam” (Gomez & Gerken, 1999; Gómez & Gerken, 2000; Saffran, 2002; Saffran et al., 2008). After exposure to habituation strings, infants discriminate AG consistent from violation sequences and generalize to novel AG consistent sequences not presented during exposure. Thus, infants manage with considerable variability in sequencing regularities and extract dependencies beyond specific words or positions.

### 4.1 Animals

In a comparative study, tamarin (New World) monkeys and infants were exposed to strings of syllables that were arranged in various patterns of different length, based on the same underlying AG (Saffran et al., 2008). When tested, the tamarins relied mainly on recognizing identical sequences shared between the test strings and strings heard during exposure. They were not able to generalize the sequencing regularities to novel AG consistent strings not heard during the exposure phase. A similar finding has also been obtained with pigeons trained with strings consisting of visual items (Herbranson & Shimp, 2008).

A study with marmosets (New World) and macaques (Old World) monkeys used a modified version of the Saffran et al. (2008) paradigm (Wilson et al., 2013), implementing different levels of sequencing complexity, including adjacent and nonadjacent dependencies. There was also a simple rule, that every legal sequence started with a specific nonsense word “A” (see Fig. 1 lower right) so any other nonsense word starting a sequence would be a simple acoustically driven rule “violation." Both marmosets and macaques were sensitive to this property. For other sequences, the marmosets responded primarily to similarities between exposure and test sequences (Wilson et al., 2013). However, the macaques showed sensitivity to the variation in adjacent regularities throughout the sequences and their responses generalized to novel testing sequences. In follow up work, the macaque behavior was compared to that of adult humans, showing that both species tracked the variability in statistical dependencies between adjacent items throughout the sequences (Wilson et al., 2015). However, neither macaques nor many humans showed sensitivity to the nonadjacent dependencies also present in this AG. A further study using the same AG in humans and macaques with auditory or visual sequences found that the two species were largely similar in sensitivity to the variability in adjacent statistical dependencies, and also that inputs from the auditory or visual modalities produced similar outcomes (Milne et al., 2017). Other macaque work using even greater variability in statistical dependencies also shows successful learning and generalization of adjacent sequencing relationships (Heimbauer et al., 2018).

### 4.2 Summary

With AGs containing variability in item transitions, pigeon and New World monkey results can be explained by relying on recognizing familiar sequences based on acoustical similarity or position cues. Macaques and human adults and infants can manage with the variability in adjacent dependencies.

## 5 Rule generalization: Marcus‐type grammars

Another grammar, initially used by Marcus et al. (1999), implements a rule‐based pattern of three item long “X” and “Y” item sequences (XYX, XYY etc; where X and Y are arbitrary items). An important aspect of this paradigm is generalization of rule learning to novel items not previously experienced.

In the seminal study, 7‐month‐old infants were habituated to XYX or XYY strings (Marcus et al., 1999). X and Y were different speech syllables, resulting in strings like “ga‐ti‐ga” or “ga‐ti‐ti” (see Fig. 1). The infants’ responses generalized to novel syllable strings, suggesting that they extracted the structural regularity, although a caveat with experiments using this grammar is that both human infants (Gerken, 2006; Kovács, 2014) and adults (Chen et al., 2015) tend to classify strings based on simpler cues, such as item identity or item repetition (“XX”) whenever such cues are available.

### 5.1 Animals

Rhesus macaques (Hauser & Glynn, 2009) habituated with XXY or XYY strings consisting of different macaque vocalizations showed dishabituation to novel strings. However, as test and habituation strings consisted of the same call types, generalization can be based on acoustical similarity.

Using a discrimination paradigm, rats were trained to distinguish XYX, XXY, and YXX strings of tones or light on/off sequences (Murphy et al., 2008). When tones were transposed to different frequencies, the rats continued to discriminate the patterns. Whether this represents robust generalization or generalization of tonal similarities is debated (Corballis, 2009; Mondragón et al., 2009).

Zebra finches (van Heijningen et al., 2013) and Bengalese finches (Seki et al., 2013) trained to discriminate XYX from XXY and XYY strings consisting of song or call elements, responded to test strings with rearranged elements by noticing the position of familiar X and Y elements, rather than the structural similarity. In another study, zebra finches and budgerigars were trained to discriminate a set of XYX from XXY strings consisting of song elements or tones (Spierings & ten Cate, 2016). The birds were then tested with strings in the same patterns, either consisting of rearrangements of familiar items or consisting of entirely novel items, bearing no similarity to familiar ones, as in the initial infant study (Marcus et al., 1999). The zebra finches classified test strings of familiar items based on similarity to the training strings, and they did not generalize the sequencing rules to the strings consisting of novel items. In stark contrast, the budgerigars classified all test strings by their sequencing structure (Spierings & ten Cate, 2016).

### 5.2 Summary

Several species rely on acoustical similarities between training and test strings to generalize Marcus‐type grammars. However, the fact that budgerigars can generalize to novel stimuli based on grammatical similarity with training stimuli indicates that this ability can be present, and they can be found in certain species.

## 6 Nonadjacent dependencies

Nonadjacent dependencies (NADs; see Fig. 1) are relationships between items separated by intervening items. Compared to adjacent dependencies, such dependencies increase working memory demands. Infants manage with NADs in their first year (Gómez & Maye, 2005), in concert with learning of hierarchical dependencies in language.

Human adults tested with an AX^+^B grammar, in which A and B items (each being a specific item, e.g., syllable) are associated in time over a number of intervening X syllables (i.e., an arbitrary item belonging to a larger set), detected the NADs with speech (Newport & Aslin, 2004) or tone sounds (Creel et al., 2004). Human NAD learning is assisted by acoustical cues identifying the NADs, for example, consonants in both A and B positions over intervening X vowels (Gómez & Maye, 2005; Lany & Gómez, 2008; Onnis et al., 2005; Wilson et al., 2018). NAD learning without acoustical cues is possible but quite variable, particularly when adjacent relationships uninformative on the NADs tax attention and memory (Fig. 1).

### 6.1 Animals

Newport and colleagues (Newport et al., 2004) exposed tamarins to the NAD strings used in their human study. The tamarins detected NADs, showing sensitivity to the NADs across vowels but not across consonants. In another study, squirrel monkeys detected NADs in tone sequences where the first and last tone were of similar pitch, separated by 1–5 repetitions of a tone at a different frequency (Ravignani et al., 2013).

Chimpanzee NAD sensitivity was tested using six‐item‐long sequences (Endress et al., 2010), in which the intervening items between A and B and the end positions were of type X. The chimpanzees relied on positional information and did not show a sensitivity to the NAD violations in which B preceded A. In another study, chimpanzees detected NADs among identical visual symbols (AX^+^A) presented simultaneously on the screen (Sonnweber et al., 2015). Exposure to nonsymmetrical dependencies (AXB, CXD, etc.) resulted in learning of which items could occupy first and the final edge positions but not their linkage.

An initial study in rats failed to find NAD learning (Toro & Trobalón, 2005). However, a later study comparing rats and humans showed that rats could discriminate consonant‐vowel (CVCVCV) sequences in which the NADs were instantiated over either the vowels or consonants (de la Mora & Toro, 2013) while humans detected the NAD only over the vowels.

The above mentioned zebra finch neuronal recording study by Lu and Vicario (2014) also tested NADs using an AXB paradigm. The study showed that auditory neurons responded differently to an item at the end of the sequences depending on whether there was a matching or nonmatching item in the first position. In another study, zebra finches were trained to detect AXB and CXD dependencies (Chen & ten Cate, 2017). The birds were sensitive to the dependencies over 1–3 repeated X‐items and maintained this sensitivity with a novel X item.

### 6.2 Summary

Noticing NADs by humans and other animals is facilitated when the dependencies are at the sequence edges, are symmetrical or share perceptual features (Wilson et al., 2018). The X number and set size are crucial for NAD generalization in infants (Gómez, 2002), but they require further exploration in nonhuman animals. It seems likely that species use different strategies to detect the nonadjacent dependencies (acoustical cues or other properties), suggesting that the processes for detection may differ. However, once detected, the cognitive operations or neural correlates of the NAD may well be similar across the species (Wilson et al., 2017, 2018).

## 7 Hierarchical dependencies

Language contains hierarchical dependencies between words and phrases. Human learning of hierarchical dependencies in AGs is challenging because the meaningless items used are difficult to remember (Perruchet & Rey, 2005). Given sufficient training, adult humans can learn nested dependencies, such as A1‐A2‐B2‐B1 where particular A's are associated with particular Bs (Bahlmann et al., 2008) or crossed‐dependencies A1‐A2‐B1‐B2 (Udden et al., 2012). Processing such dependencies requires combinatorial capacities that can manage with multiple dependencies, and hence are more complex than the grammars considered thus far (Fig. 1).

### 7.1 Animals

Fitch and Hauser (2004) habituated tamarins and adult humans to speech syllables drawn from two categories of sounds (A or B) produced by male or female human speakers. The syllables were arranged to contain adjacent (AB)^n^ dependencies (ABAB; ABABAB) or followed an A^n^B^n^ rule (AABB; AAABBB). The A^n^B^n^ pattern requires matching the number of A and B items, although not their relation (i.e., A1‐B1 and A2‐B2). Humans distinguished correct and violation sequences from either grammar; however, the tamarins only noticed the adjacent (AB)^n^ dependencies.

In a self‐training paradigm with touch screens (Rey et al., 2012), baboons learned pairwise associations between visual symbols (A1‐B1, A2‐B2, etc.). In testing, the baboons observed the initial A items of two pairs (A1‐A2) and were then required to select the matching items for reward. The animals preferentially paired the B partner of the most recently observed A item (e.g., A2‐B2), followed by the partner pair of the first element (e.g., A1‐B1), thereby most often selecting the pattern: A1‐A2‐B2‐B1. Although this resembles a hierarchical center‐embedded structure, the authors note that the baboons can rely on an associative memory trace pairing the correct As and Bs, not requiring hierarchical organization of dependencies (see also Poletiek et al., 2016).

Two songbird species have also been trained with ABAB versus AABB strings: starlings (Gentner et al., 2006) and zebra finches (van Heijningen et al., 2009). For the starlings the A’s and B’s consisted of two starling song phrases and for the zebra finches two types of song elements. Both species distinguished (AB)^2^ from A^2^B^2^. However, most zebra finches failed to generalize the A^n^B^n^ pattern to new item types and attended to adjacent regularities in training strings (such as AA bigrams) rather than the full structure (van Heijningen et al., 2009). Whether this also holds for the starlings is unknown. Pigeons and keas (a parrot) trained to recognize visual shapes in (AB)^n^ or A^n^B^n^ sequences (Stobbe et al., 2012) also relied on adjacent bigram dependencies. The keas all attended to BA transitions, while the pigeons showed an idiosyncratic mix of strategies.

A recent human infant and macaque study on sequencing and rule reversal using a mirror grammar (ABC‐CBA) showed that both species can process the sequence and reverse rules (Jiang et al., 2018), which by linguistic definition is more complex (“supra‐regular”) but whether the learning depends on hierarchically organized processes is unclear (see also Fountain & Rowan, 1995).

### 7.2 Summary

So far, the available nonhuman animal experiments do not provide unambiguous evidence of hierarchically organized structure learning. However, this does not mean that no animal is capable of hierarchical processing; the training stimuli used so far could all be discriminated by using local (adjacent) sequencing dependencies or acoustical cues. Even humans require considerable training to learn complex nested or crossed dependencies (Udden et al., 2012), likely because AGL tasks lack semantic relationships. This area of AGL research remains controversial but important for study.

## 8 Animal‐structured sequence learning, cognitive substrates, and language evolution

Language consists of flexible semantic categories that are readily combined and can be hierarchically organized using syntactic knowledge. Language learning is, however, not immediate. It takes time to master the ability to create virtually unbounded expressive communication, the likes of which is not seen in nonhuman animals. Some of the core combinatorial processes, particularly those that infants learn to manage with early in their first year of life as their language skills improve appear to have been evolutionarily conserved, and thus are not specific to language or unique to humans.

Overall, all species tested thus far can detect certain types of adjacent dependencies between items in a sequence. Santolin & Saffran (2017) suggest that statistical learning is a general mechanism for forming memory traces that becomes the foundation for other cognitive operations. The empirical evidence shows that many animals (humans included) will rely on adjacent co‐occurrences, acoustical or positional cues whenever possible. Pigeon, marmoset, and tamarin results, for instance, can be explained by attending to acoustical or positional cues. At the same time, acoustical cues such as the frequency of two or more items co‐occurring, repetition of the same acoustical item, positional information, or silent gaps between syllables can also benefit animals and human infants (Gerken, 2006) detection of sequencing patterns.

However, although part of the generalizations observed in nonhuman animal AGL data might be explained by animals attending to acoustical similarities (Beckers et al., 2016) or familiar substrings rather than by learning sequencing dependencies, other data cannot, particularly when item and position cues become unreliable. For instance, Reber‐type grammars contain considerable variability between item transitions, and macaques and humans show sensitivity to the variability in the statistical cues for adjacent dependencies. Also, results showing generalization of the structure of sequencing patterns to entirely novel sounds, such as by budgerigars in the Marcus‐type grammars, cannot be explained by acoustical similarity or physical cues. Moreover, given the large variability in species, experimental paradigms and stimuli that have been used so far, more extensive cross‐species comparisons are needed to better identify animal abilities, to detect where an animal’s limits lie regarding structured sequence learning and to deduce the evolutionary pattern (Fig. 1).

Briefly, with respect to the neural operations underlying the various types of processing, neurobiological studies have begun to study cross‐species correspondences and specializations in brain functions for some operations (Petkov & Marslen‐Wilson, 2018). Neural processes that adapt to sensory stimulus repetition are nearly ubiquitous in animal brains and serve as predictive signals for already experienced events (Friston, 2010). Temporal regularities in sounds, neuronal properties, and auditory working memory capacity altogether determine and constrain how mammals perceive auditory objects and sequences. The promising results obtained so far call for a further exploration of the core neural processes and mechanisms involved in sequence learning.

Theoretically, neural substrates that support language‐specific operations or cognitive functions more generally, including sequencing knowledge, are thought to rely on a broader relational knowledge system (Halford et al., 2010; Shanks, 2010) that allows us to combine, rank, establish causality, and flexibly manipulate information. Thereby, as examples, monkey sequencing rule‐reversal or the learning of multiple longer‐range associations (A1 to B1 with A2‐B2 intervening, see above) could be informative particularly when such operations are compared to those for hierarchical language processes in humans. This underscores the ability of human research to distinguish language‐specific from domain‐general processes and for comparative work with nonhuman animals to identify which domain general processes are also evolutionarily conserved.

There is considerable discussion about the aspects of learning and cognition that are implicit (not requiring perceptual awareness and dependent on a procedural learning system) or explicit (requiring perceptual awareness and hippocampal memory circuit dependent), which is relevant to understanding impairments of cognition and neural systems. Whether or not animals are aware of having learning rules is difficult to establish. However, their behavior can reveal the kind of sequential construction they can make, and a better understanding of animal‐structured sequence learning behavior will provide a vital foundation for understanding neural mechanisms.

## 9 Conclusions

Structured sequence learning tasks can be used to emulate various combinatorial operations in a multidimensional space of sequencing complexity (Fig. 1), allowing assessment of the form and extent of the combinatorial learning capacities of different nonhuman animals. While sequence processing abilities are clearly present, it is still too early to draw firm conclusions about how certain abilities differ, evolved, and gave rise to the co‐evolutionary interactions between language and cognition in humans (Jackendoff & Wittenberg, 2017; Schulze et al., 2012). Studies in nonhuman animals are still catching up with experiments in human infants and the field of cognitive science will benefit from studying a broader range of species. Moreover, there is a great need to use more naturalistic grammar learning tasks, because even humans struggle to learn complex hierarchical sequencing dependencies in tasks devoid of meaning. The good news is that we have yet to understand the limits of nonhuman animal cognitive abilities, the pursuit of which will greatly illuminate how language‐related combinatorial capacities evolved.

## References

[tops12444-bib-0001] Aslin, R. N. , Saffran, J. R. , & Newport, E. L. (1998). Computation of conditional probability statistics by 8‐month‐old infants. Psychological Science, 9(4), 321–324.

[tops12444-bib-0002] Bahlmann, J. , Schubotz, R. I. , & Friederici, A. D. (2008). Hierarchical artificial grammar processing engages Broca's area. NeuroImage, 42(2), 525–534. 18554927 10.1016/j.neuroimage.2008.04.249

[tops12444-bib-0003] Beckers, G. J. L. , Berwick, R. C. , Okanoya, K. , & Bolhuis, J. J. (2016). What do animals learn in artificial grammar studies? Neuroscience & Biobehavioral Reviews, 81, 238–246. 28017840 10.1016/j.neubiorev.2016.12.021

[tops12444-bib-0004] Chen, J. , & ten Cate, C. (2015). Zebra finches can use positional and transitional cues to distinguish vocal element strings. Behavioural Processes, 117, 29–34. 25217867 10.1016/j.beproc.2014.09.004

[tops12444-bib-0005] Chen, J. , & ten Cate, C. (2017). Bridging the gap: Learning of acoustic nonadjacent dependencies by a songbird. Journal of Experimental Psychology: Animal Learning and Cognition, 43(3), 295‐302. 29120216 10.1037/xan0000145

[tops12444-bib-0006] Chen, J. , van Rossum, D. , & ten Cate, C. (2015). Artificial grammar learning in zebra finches and human adults: XYX versus XXY. Animal Cognition, 18(1), 151–164. 25015135 10.1007/s10071-014-0786-4

[tops12444-bib-0007] Comins, J. A. , & Gentner, T. Q. (2010). Working memory for patterned sequences of auditory objects in a songbird. Cognition, 117(1), 38–53. 20638052 10.1016/j.cognition.2010.06.009 PMC2934891

[tops12444-bib-0008] Corballis, M. C. (2009). Do rats learn rules? Animal Behaviour, 78(4), e1–e2.

[tops12444-bib-0009] Creel, S. C. , Newport, E. L. , & Aslin, R. N. (2004). Distant melodies: Statistical learning of nonadjacent dependencies in tone sequences. Journal of Experimental Psychology: Learning, Memory, and Cognition, 30(5), 1119. 10.1037/0278-7393.30.5.1119 15355140

[tops12444-bib-0010] de la Mora, M. , & Toro, J. M. (2013). Rule learning over consonants and vowels in a non‐human animal. Cognition, 126(2), 307–312. 23121712 10.1016/j.cognition.2012.09.015 PMC4217073

[tops12444-bib-0011] Dehaene, S. , Meyniel, F. , Wacongne, C. , Wang, L. , & Pallier, C. (2015). The neural representation of sequences: From transition probabilities to algebraic patterns and linguistic trees. Neuron, 88(1), 2–19. 26447569 10.1016/j.neuron.2015.09.019

[tops12444-bib-0012] Endress, A. D. , Carden, S. , Versace, E. , & Hauser, M. D. (2010). The apes’ edge: Positional learning in chimpanzees and humans. Animal Cognition, 13(3), 483–495. 20012457 10.1007/s10071-009-0299-8

[tops12444-bib-0013] Fitch, W. T. , Friederici, A. D. , & Hagoort, P. (2012). Pattern perception and computational complexity: Introduction to the special issue. Philosophical Transactions of the Royal Society B: Biological Sciences, 367(1598), 1925–1932. 10.1098/rstb.2012.0099 PMC3367691 22688630

[tops12444-bib-0014] Fitch, W. T. , & Hauser, M. D. (2004). Computational constraints on syntactic processing in a nonhuman primate. Science, 303(5656), 377–380. 14726592 10.1126/science.1089401

[tops12444-bib-0015] Fountain, S. B. , & Rowan, J. D. (1995). Coding of hierarchical versus linear pattern structure in rats and humans. Journal of Experimental Psychology: Animal Behavior Processes, 21(3), 187–202. 7602257 10.1037//0097-7403.21.3.187

[tops12444-bib-0016] Friston, K. (2010). The free‐energy principle: A unified brain theory? Nature Reviews Neuroscience, 11(2), 127–138. 20068583 10.1038/nrn2787

[tops12444-bib-0017] Gentner, T. Q. , Fenn, K. M. , Margoliash, D. , & Nusbaum, H. C. (2006). Recursive syntactic pattern learning by songbirds. Nature, 440(7088), 1204–1207. 16641998 10.1038/nature04675 PMC2653278

[tops12444-bib-0018] Gerken, L. (2006). Decisions, decisions: Infant language learning when multiple generalizations are possible. Cognition, 98(3), B67–B74. 15992791 10.1016/j.cognition.2005.03.003

[tops12444-bib-0019] Gervain, J. , de la Cruz‐Pavia, I. , & Gerken, L. (2018). Behavioral and imaging studies of infant artificial grammar learning. Topics in Cognitive Science, epub ahead of print: 10.1111/tops.12400. 30554481

[tops12444-bib-0020] Gervain, J. , & Mehler, J. (2010). Speech perception and language acquisition in the first year of life. Annual Review of Psychology, 61, 191–218. 10.1146/annurev.psych.093008.100408 19575623

[tops12444-bib-0021] Gómez, R. L. (2002). Variability and detection of invariant structure. Psychological Science, 13(5), 431–436. 12219809 10.1111/1467-9280.00476

[tops12444-bib-0022] Gomez, R. L. , & Gerken, L. (1999). Artificial grammar learning by 1‐year‐olds leads to specific and abstract knowledge. Cognition, 70(2), 109–135. 10349760 10.1016/s0010-0277(99)00003-7

[tops12444-bib-0023] Gómez, R. L. , & Gerken, L. (2000). Infant artificial language learning and language acquisition. Trends in Cognitive Sciences, 4(5), 178–186. 10782103 10.1016/s1364-6613(00)01467-4

[tops12444-bib-0024] Gómez, R. , & Maye, J. (2005). The developmental trajectory of nonadjacent dependency learning. Infancy, 7(2), 183–206. 10.1207/s15327078in0702_4 33430549

[tops12444-bib-0025] Halford, G. S. , Wilson, W. H. , & Phillips, S. (2010). Relational knowledge: The foundation of higher cognition. Trends in Cognitive Sciences, 14(11), 497–505. 20884275 10.1016/j.tics.2010.08.005

[tops12444-bib-0026] Hauser, M. D. , & Glynn, D. (2009). Can free‐ranging rhesus monkeys (Macaca mulatta) extract artificially created rules comprised of natural vocalizations? Journal of Comparative Psychology, 123(2), 161–167. 19450023 10.1037/a0015584

[tops12444-bib-0027] Hauser, M. D. , Newport, E. L. , & Aslin, R. N. (2001). Segmentation of the speech stream in a non‐human primate: Statistical learning in cotton‐top tamarins. Cognition, 78(3), B53–64. 11124355 10.1016/s0010-0277(00)00132-3

[tops12444-bib-0028] Heimbauer, L. A. , Conway, C. M. , Christiansen, M. H. , Beran, M. J. , & Owren, M. J. (2018). Visual artificial grammar learning by rhesus macaques (Macaca mulatta): exploring The role of grammar complexity and sequence length. Animal Cognition, 21(2), 267–284. 29435770 10.1007/s10071-018-1164-4

[tops12444-bib-0029] Herbranson, W. T. , & Shimp, C. P. (2008). Artificial grammar learning in pigeons. Learning & Behavior, 36(2), 116–137. 18543712 10.3758/lb.36.2.116

[tops12444-bib-0030] Jackendoff, R. , & Wittenberg, E. (2017). Linear grammar as a possible stepping‐stone in the evolution of language. Psychonomic Bulletin & Review, 24(1), 219–224. 27368633 10.3758/s13423-016-1073-y

[tops12444-bib-0031] Jaeger, G. , & Rogers, J. (2012). Formal language theory: Refining the Chomsky hierarchy. Philosophical Transactions of the Royal Society of London. Series B, Biological sciences, 367, 1956–1970. 22688632 10.1098/rstb.2012.0077 PMC3367686

[tops12444-bib-0032] Jiang, X. , Long, T. , Cao, W. , Li, J. , Dehaene, S. , & Wang, L. (2018). Production of supra‐regular spatial sequences by macaque monkeys. Current Biology, 28(12), 1851–1859. 29887304 10.1016/j.cub.2018.04.047 PMC6606444

[tops12444-bib-0033] Kovács, Á. M. (2014). Extracting regularities from noise: Do infants encode patterns based on same and different relations? Language Learning, 64(s2), 65–85.

[tops12444-bib-0034] Lany, J. , & Gómez, R. L. (2008). Twelve‐month‐old infants benefit from prior experience in statistical learning. Psychological Science, 19(12), 1247–1252. 19121132 10.1111/j.1467-9280.2008.02233.x PMC2967014

[tops12444-bib-0035] Lu, K. , & Vicario, D. S. (2014). Statistical learning of recurring sound patterns encodes auditory objects in songbird forebrain. Proceedings of the National Academy of Sciences, 111(40), 14553–14558. 10.1073/pnas.1412109111 PMC4210023 25246563

[tops12444-bib-0036] Marcus, G. F. , Vijayan, S. , Bandi Rao, S. , & Vishton, P. M. (1999). Rule learning by seven‐month‐old infants. Science, 283(5398), 77–80. 9872745 10.1126/science.283.5398.77

[tops12444-bib-0037] Milne, A. E. , Petkov, C. I. , & Wilson, B. (2017). Auditory and visual sequence learning in humans and monkeys using an artificial grammar learning paradigm. Neuroscience, 389, 104–117. 28687306 10.1016/j.neuroscience.2017.06.059 PMC6278909

[tops12444-bib-0038] Mondragón, E. , Murphy, R. A. , & Murphy, V. A. (2009). Rats do learn XYX rules. Animal Behaviour, 78(4), e3–e4.

[tops12444-bib-0039] Mueller, J. L. , ten Cate, C. , & Toro, J. M. (2018). A comparative perspective on the role of acoustic cues in detecting language structure. Topics in Cognitive Science. epub ahead of print: 10.1111/tops.12373. 30033636

[tops12444-bib-0040] Murphy, R. A. , Mondragon, E. , & Murphy, V. A. (2008). Rule learning by rats. Science, 319(5871), 1849–1851. 18369151 10.1126/science.1151564

[tops12444-bib-0041] Newport, E. L. , & Aslin, R. N. (2004). Learning at a distance I. Statistical learning of non‐adjacent dependencies. Cognitive Psychology, 48(2), 127–162. 14732409 10.1016/s0010-0285(03)00128-2

[tops12444-bib-0042] Newport, E. L. , Hauser, M. D. , Spaepen, G. , & Aslin, R. N. (2004). Learning at a distance II. Statistical learning of non‐adjacent dependencies in a non‐human primate. Cognitive Psychology, 49(2), 85–117. 15304368 10.1016/j.cogpsych.2003.12.002

[tops12444-bib-0043] Onnis, L. , Monaghan, P. , Richmond, K. , & Chater, N. (2005). Phonology impacts segmentation in online speech processing. Journal of Memory and Language, 53(2), 225–237.

[tops12444-bib-0044] Perruchet, P. , & Rey, A. (2005). Does the mastery of center‐embedded linguistic structures distinguish humans from nonhuman primates? Psychonomic Bulletin & Review, 12(2), 307–313. 16082811 10.3758/bf03196377

[tops12444-bib-0045] Petkov, C. I. , & Marslen‐Wilson, W. D. (2018). Editorial overview: The evolution of language as a neurobiological system. Current Opinion in Behavioral Sciences, 21, v–xii. 30057938 10.1016/j.cobeha.2018.06.002 PMC6058084

[tops12444-bib-0046] Petkov, C. I. , & Wilson, B. (2012). On the pursuit of the brain network for proto‐syntactic learning in non‐human primates: Conceptual issues and neurobiological hypotheses. Philosophical Transactions of the Royal Society of London. Series B, Biological sciences, 367(1598), 2077–2088. 22688642 10.1098/rstb.2012.0073 PMC3367685

[tops12444-bib-0047] Poletiek, F. H. , Fitz, H. , & Bocanegra, B. R. (2016). What baboons can (not) tell us about natural language grammars. Cognition, 151, 108–112. 26026382 10.1016/j.cognition.2015.04.016

[tops12444-bib-0048] Ravignani, A. , Sonnweber, R.‐S. , Stobbe, N. , & Fitch, W. T. (2013). Action at a distance: Dependency sensitivity in a New World primate. Biology Letters, 9(6), 20130852. 24227047 10.1098/rsbl.2013.0852 PMC3871375

[tops12444-bib-0049] Reber, A. S. (1967). Implicit learning of artificial grammars. Journal of Verbal Learning and Verbal Behaviour, 6(6), 855–863.

[tops12444-bib-0050] Rey, A. , Perruchet, P. , & Fagot, J. (2012). Centre‐embedded structures are a by‐product of associative learning and working memory constraints: Evidence from baboons (Papio Papio). Cognition, 123(1), 180–184. 22225966 10.1016/j.cognition.2011.12.005

[tops12444-bib-0051] Saffran, J. R. (2002). Constraints on statistical language learning. Journal of Memory and Language, 47(1), 172–196.

[tops12444-bib-0052] Saffran, J. R. , Aslin, R. N. , & Newport, E. L. (1996). Statistical learning by 8‐month‐old infants. Science, 274(5294), 1926–1928. 8943209 10.1126/science.274.5294.1926

[tops12444-bib-0053] Saffran, J. , Hauser, M. , Seibel, R. , Kapfhamer, J. , Tsao, F. , & Cushman, F. (2008). Grammatical pattern learning by human infants and cotton‐top tamarin monkeys. Cognition, 107(2), 479–500. 18082676 10.1016/j.cognition.2007.10.010 PMC2386981

[tops12444-bib-0054] Santolin, C. , & Saffran, J. R. (2017). Constraints on statistical learning across species. Trends in Cognitive Sciences, 22(1), 52–63. 29150414 10.1016/j.tics.2017.10.003 PMC5777226

[tops12444-bib-0055] Schlenker, P. , Chemla, E. , & Zuberbühler, K. (2016). What do monkey calls mean? Trends in Cognitive Sciences, 20(12), 894–904. 27836778 10.1016/j.tics.2016.10.004

[tops12444-bib-0056] Schulze, K. , Vargha‐Khadem, F. , & Mishkin, M. (2012). Test of a motor theory of long‐term auditory memory. Proceedings of the National Academy of Sciences, 109(18), 7121–7125. 10.1073/pnas.1204717109 PMC3345014 22511719

[tops12444-bib-0057] Seki, Y. , Suzuki, K. , Osawa, A. M. , & Okanoya, K. (2013). Songbirds and humans apply different strategies in a sound sequence discrimination task. Frontiers in Psychology, 4, 447. 23882247 10.3389/fpsyg.2013.00447 PMC3713397

[tops12444-bib-0058] Shanks, D. R. (2010). Learning: From association to cognition. Annual Review of Psychology, 61, 273–301. 10.1146/annurev.psych.093008.100519 19575617

[tops12444-bib-0059] Sonnweber, R. , Ravignani, A. , & Fitch, W. T. (2015). Non‐adjacent visual dependency learning in chimpanzees. Animal Cognition, 18(3), 733–745. 25604423 10.1007/s10071-015-0840-x PMC4412729

[tops12444-bib-0060] Spierings, M. , de Weger, A. , & ten Cate, C. (2015). Pauses enhance chunk recognition in song element strings by zebra finches. Animal Cognition, 18(4), 867–874. 25771964 10.1007/s10071-015-0855-3 PMC4460290

[tops12444-bib-0061] Spierings, M. J. , & ten Cate, C. (2016). Budgerigars and zebra finches differ in how they generalize in an artificial grammar learning experiment. Proceedings of the National Academy of Sciences, 113(27), E3977–E3984. 10.1073/pnas.1600483113 PMC4941449 27325756

[tops12444-bib-0062] Stobbe, N. , Westphal‐Fitch, G. , Aust, U. , & Fitch, W. T. (2012). Visual artificial grammar learning: Comparative research on humans, kea (Nestor notabilis) and pigeons (Columba livia). Philosophical Transactions of the Royal Society B: Biological Sciences, 367(1598), 1995–2006. 10.1098/rstb.2012.0096 PMC3367688 22688635

[tops12444-bib-0063] ten Cate, C. , & Okanoya, K. (2012). Revisiting the syntactic abilities of non‐human animals: Natural vocalizations and artificial grammar learning. Philosophical Transactions of the Royal Society of London. Series B, Biological Sciences, 367, 1984–1994. 22688634 10.1098/rstb.2012.0055 PMC3367684

[tops12444-bib-0064] Toro, J. , & Trobalón, J. (2005). Statistical computations over a speech stream in a rodent. Perception & Psychophysics, 67(5), 867–875. 16334058 10.3758/bf03193539

[tops12444-bib-0065] Udden, J. , Ingvar, M. , Hagoort, P. , & Petersson, K. M. (2012). Implicit acquisition of grammars with crossed and nested non‐adjacent dependencies: Investigating the push‐down stack model. Cognitive Science, 36(6), 1078–1101. 22452530 10.1111/j.1551-6709.2012.01235.x

[tops12444-bib-0066] van Heijningen, C. A. A. , Chen, J. , van Laatum, I. , van der Hulst, B. , & ten Cate, C. (2013). Rule learning by zebra finches in an artificial grammar learning task: Which rule? Animal cognition, 16(2), 165–175. 22971840 10.1007/s10071-012-0559-x

[tops12444-bib-0067] van Heijningen, C. A. , de Visser, J. , Zuidema, W. , & ten Cate, C. (2009). Simple rules can explain discrimination of putative recursive syntactic structures by a songbird species. Proceedings of the National Academy of Sciences USA, 106(48), 20538–20543. 10.1073/pnas.0908113106 PMC2787117 19918074

[tops12444-bib-0068] Wilson, B. , Marslen‐Wilson, W. D. , & Petkov, C. I. (2017). Conserved Sequence Processing in Primate Frontal Cortex. Trends in Neurosciences, 40(2), 72–82. 28063612 10.1016/j.tins.2016.11.004 PMC5359391

[tops12444-bib-0069] Wilson, B. , Slater, H. , Kikuchi, Y. , Milne, A. E. , Marslen‐Wilson, W. D. , Smith, K. , & Petkov, C. I. (2013). Auditory artificial grammar learning in macaque and marmoset monkeys. Journal of Neuroscience, 33(48), 18825–18835. 24285889 10.1523/JNEUROSCI.2414-13.2013 PMC3841451

[tops12444-bib-0070] Wilson, B. , Smith, K. , & Petkov, C. I. (2015). Mixed‐complexity artificial grammar learning in humans and macaque monkeys: Evaluating learning strategies. European Journal of Neuroscience, 41(5), 568–578. 25728176 10.1111/ejn.12834 PMC4493314

[tops12444-bib-0071] Wilson, B. , Spierings, M. , Ravignani, A. , Mueller, J. L. , Mintz, T. H. , Wijnen, F. , van der Kant, A. , Smith, K. , & Rey, A. (2018). Non‐adjacent dependency learning in humans and other animals. Topics in Cognitive Science, epub ahead of print: 10.1111/tops.12381 PMC7496455 32729673

